# Functional and structural gradients reveal atypical hierarchical organization of Parkinson's disease

**DOI:** 10.1002/hbm.26647

**Published:** 2024-03-15

**Authors:** Jinglong Wu, Lihua Ma, Di Luo, Zhaohui Jin, Li Wang, Luyao Wang, Ting Li, Jian Zhang, Tiantian Liu, Diyang Lv, Tianyi Yan, Boyan Fang

**Affiliations:** ^1^ School of Mechatronical Engineering, Beijing Institute of Technology Beijing China; ^2^ Parkinson Medical Center, Beijing Rehabilitation Hospital, Capital Medical University Beijing China; ^3^ School of Life Science, Beijing Institute of Technology Beijing China; ^4^ School of Life Science, Shanghai University Shanghai China

**Keywords:** functional gradient, magnetic resonance imaging, Parkinson's disease, structural gradient

## Abstract

Parkinson's disease (PD) patients exhibit deficits in primary sensorimotor and higher‐order executive functions. The gradient reflects the functional spectrum in sensorimotor‐associated areas of the brain. We aimed to determine whether the gradient is disrupted in PD patients and how this disruption is associated with treatment outcome. Seventy‐six patients (mean age, 59.2 ± 12.4 years [standard deviation], 44 women) and 34 controls participants (mean age, 58.1 ± 10.0 years [standard deviation], 19 women) were evaluated. We explored functional and structural gradients in PD patients and control participants. Patients were followed during 2 weeks of multidisciplinary intensive rehabilitation therapy (MIRT). The Unified Parkinson's Disease Rating Scale Part III (UPDRS‐III) was administered to patients before and after treatment. We investigated PD‐related alterations in the principal functional and structural gradients. We further used a support vector machine (SVM) and correlation analysis to assess the classification ability and treatment outcomes related to PD gradient alterations, respectively. The gradients showed significant differences between patients and control participants, mainly in somatosensory and visual networks involved in primary function, and higher‐level association networks (dorsal attentional network (DAN) and default mode network (DMN)) related to motor control and execution. On the basis of the combined functional and structural gradient features of these networks, the SVM achieved an accuracy of 91.2% in discriminating patients from control participants. Treatment reduced the gradient difference. The altered gradient exhibited a significant correlation with motor improvement and was mainly distributed across the visual network, DAN and DMN. This study revealed damage to gradients in the brain characterized by sensorimotor and executive control deficits in PD patients. The application of gradient features to neurological disorders could lead to the development of potential diagnostic and treatment markers for PD.

## Graphical abstract

We are the first study to investigate somatomotor‐association impairment and MIRT‐induced brain functional and structural alterations in PD by assessing functional and structural gradients. Abnormal functional and structural gradients were distributed over networks including the primary sensorimotor network and the higher association network related to motor control and executive.

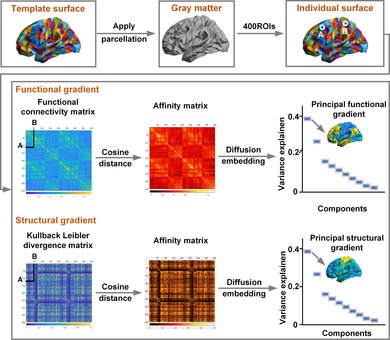

## 1 INTRODUCTION

PD is a multisystem disorder that presents with a combination of motor (Rodriguez‐Oroz et al., 2009) and nonmotor symptoms (Barone et al., 2011). Previous studies using resting‐state functional magnetic resonance imaging (rs‐fMRI) have reported abnormal changes in multiple brain networks in patients with PD (Armstrong & Okun, 2020), but the underlying neurobiological mechanism of hierarchical organization deficits in these networks remains to be elucidated.

Previous neuroimaging studies in PD patients have targeted the dysfunction of brain networks (White et al., 2020) and the abnormal characterization of network topology (Schindlbeck et al., 2021). The disrupted functional connectivity and the extensive alterations in cortical structural connectivity might cause the freezing of gait seen in PD patients (Gallea et al., 2021). Sensorimotor impairments in PD patients are related to abnormal functional connectivity (Fiorenzato et al., 2019) and disrupted network integration in the brain (Dekosky & Marek, 2003). The co‐occurrence and interplay of low‐ and high‐level network hierarchical abnormalities in PD have not been studied. A hierarchical architecture is one of the fundamental organizational principles of the brain, allowing information encoding and integration from sensation to association (Mesulam, 1998). In the present study, we combined the brain activity of primary sensorimotor processing areas with higher association areas from the perspectives of function and structure to provide a more comprehensive understanding of the pathogenesis of PD.

Researchers have emphasized the importance of understanding the whole‐brain network topology and characterized the system‐level principles of brain organization through a gradient approach (van den Heuvel & Sporns, 2019). A gradient represents a spatially continuous axis of variance in cortical features, along which brain regions are arranged. Regions that share similarities in the feature of interest occupy corresponding positions along this gradient. The significant role played by cortical location prompts us to explore a novel perspective through which we seek to comprehend the cerebral cortex in terms of its intrinsic dimensions. This gradient constitutes the fundamental dimension of an inherent coordinate system within the human cerebral cortex (Huntenburg et al., 2018) and reflects the relative differences in connectivity patterns among regions. This gradient is an important biomarker for identifying clinical diseases. Functional gradients have been proposed for major depressive disorder (Xia et al., 2022) and autism spectrum disorder (Hong et al., 2019). However, the concept of a structural gradient has not been proposed or applied to such diseases. Moreover, there is no evidence regarding whether and how the functional and structural gradients are disrupted in PD patients or whether this disruption could help predict treatment outcomes. Therefore, a combined analysis of functional and structural gradients could provide insight into the comprehensive network mechanisms, helping to elucidate the concurrence of sensorimotor impairments in PD.

In the present study, we hypothesized that there is a principal functional and structural gradient from sensorimotor to association in PD, and that this principal gradient is disrupted. Treatment may modulate the principal gradient in the primary sensorimotor involved in primary function and association networks related to motor control and executive function.

## 2 MATERIALS AND METHODS

### 2.1 Participants and clinical assessment

**FIGURE 1:**
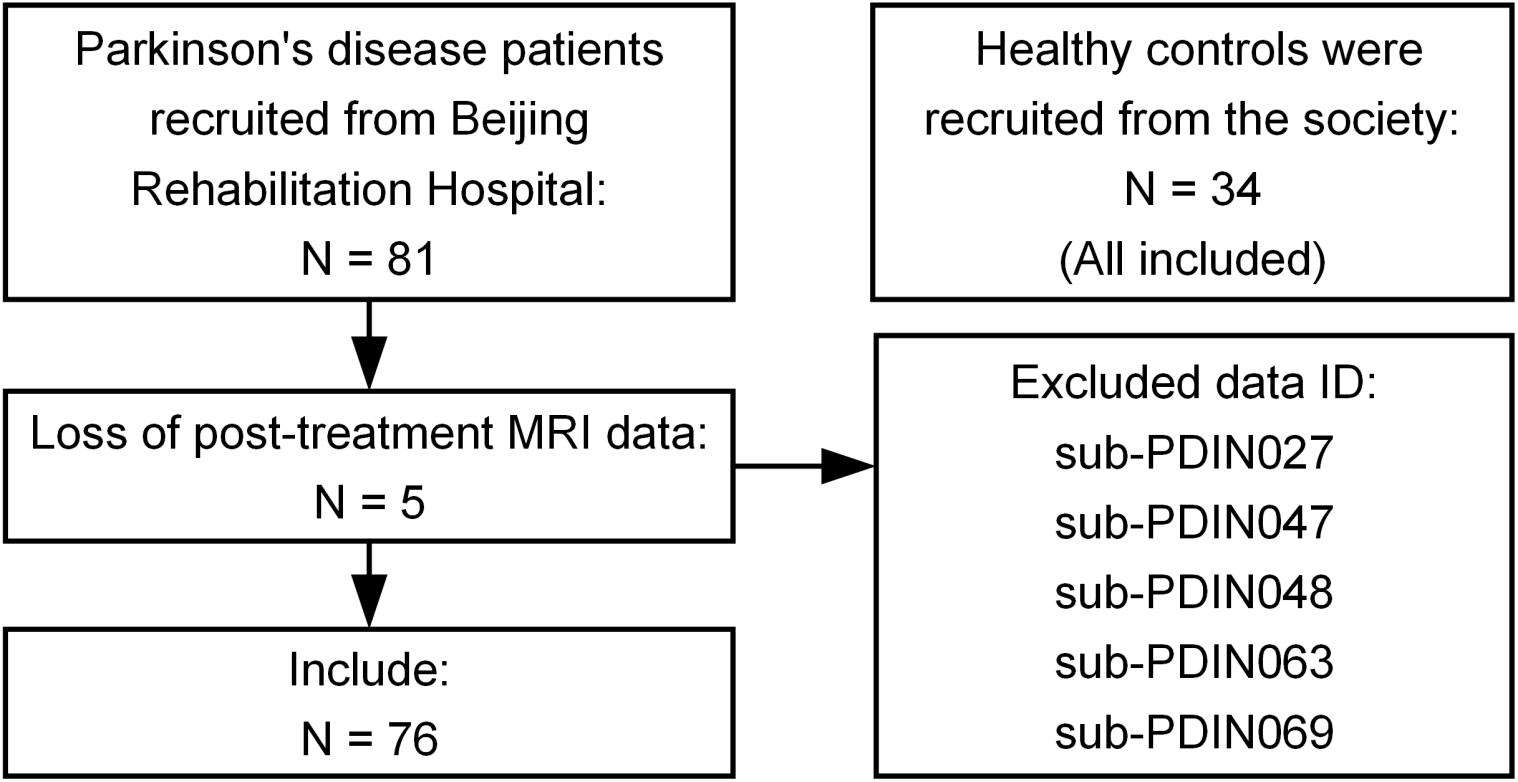
Participant flow diagram. A total of 76 participants from the Beijing Rehabilitation Hospital and 34 healthy control participants from social recruitment were included in the study.

The present study was approved by the Medical Ethics Committee of Beijing Rehabilitation Hospital. All participants provided written informed consent in accordance with the Declaration of Helsinki. For this prospective cohort study (April–November 2020, registration number ChiCTR2000033768), subjects were recruited from Beijing Rehabilitation Hospital (Table 1). All patients were diagnosed by the Movement Disorder Society diagnostic criteria in the early to middle stage of the disease. Thirty‐four healthy control participants were matched to patients in terms of age, gender, and education. The inclusion and exclusion criteria are listed in Figure 1.

**TABLE 1:** Participant demographic and clinical characteristics.

	Controls (*N* = 34)	Pre_PD (*N* = 76)	Post_PD (*N* = 76)	*p*
Age, years	58.1 ± 10.0	59.2 ± 12.4	59.2 ± 12.4	.605^a^
Sex male, *n* (%)	14 (42%)	31 (41%)	31 (41%)	.970^b^
Education, years	13.1 ± 3.8	12.9 ± 3.6	12.9 ± 3.6	.346^a^
Hoehn and Yahr stage	—	2.3 ± 0.5	2.3 ± 0.5	
Disease duration, years	—	7.0 ± 4.5	7.0 ± 4.5	
Borg, total score	—	0.8 ± 1.3	0.5 ± 0.9	**.004** ^c^
6MWT, m	—	448.7 ± 86.6	462.9 ± 89.3	**.012** ^c^
TUG, average score	—	9.9 ± 3.6	9.0 ± 2.1	**.013** ^c^
UPDRS‐III, total score	—	31.3 ± 12.1	28.8 ± 11.4	**.011** ^c^
MMSE, total score	—	27.6 ± 2.5	—	
MoCA, total score	—	25.4 ± 3.4	—	

*Note*: *p* values in bold indicate statistical significance. Values are presented as the mean ± SDs.

Abbreviations: Post_PD, PD after treatment; Pre_PD, PD before treatment.

^a^ Independent‐samples *t* test.

^b^ Chi‐square test.

^c^ Paired‐samples *t* test.

General medical and neurological examinations and semistructured interviews for obtaining medical, drug, and family histories were performed at the time of diagnosis. Motor severity, global cognition, and disease stage were determined using the UPDRS‐III Movement Disorder Society Task Force on Rating Scales for Parkinson's Disease (2003), 6‐minute walk test (6MWT), timed up and go test (TUG) (Chen, Jin, et al., 2021), Mini‐Mental State Examination (MMSE), Montreal Cognitive Assessment (MoCA), and Hoehn and Yahr scale (Li et al., 2023), respectively. All patients received MIRT (Rochester & Espay, 2015) for 2 weeks, 5 days a week, and a daily rehabilitation programme consisting of four parts was used, each part lasted 30 min–1 h in the ON state (1–2 h after medication), and treatment outcomes were recorded (Appendix S1).

### 2.2 Image acquisition and preprocessing

Patients underwent a scan more than 12 h after the withdrawal of their dopaminergic medications in a clinically defined “on‐state.” All participants were asked to close their eyes and keep their heads still. Magnetic resonance data were acquired using a 3.0 Tesla GE SIGNA Pioneer scanner. The rs‐fMRI data were acquired using a gradient‐recalled echo‐planar imaging (GRE‐EPI) sequence (TR/TE = 2000 ms/35 ms, flip angle (FA) = 90°). Thirty transverse slices (FOV = 280 × 280 mm^2^, matrix size = 128 × 128, slice thickness = 4.0 mm, slice number = 40, 250 volumes in 8 min and 20 s) were obtained. High‐resolution T1‐weighted (T1w) images were acquired using a three‐dimensional brain volume (3D BRAVO) sequence (TR/TE = 8.06/3.06 ms, FA = 15°, FOV = 300 × 300 mm^2^, matrix size = 512 × 512, slice thickness = 1.0 mm, and slice number = 160).

T1 data were preprocessed with FreeSurfer (https://surfer.nmr.mgh.harvard.edu/) and ANTs (v5.0.9, https://www.nitrc.org/projects/ants), including intensity nonuniformity correction, skull stripping, cortical extraction, and segmentation. The rs‐fMRI data were processed was performed using DPABISurf (http://www.rfmri.org/dpabi), which included removing the first 10 frames, performing slice‐timing correction, performing surface‐based smoothing (5‐mm FWHM) and performing motion correction (Appendix S2).

### 2.3 Connectivity matrix construction

The cortical surface was initially parcellated into spatially continuous cortical parcellations derived from the Schaefer400 templates (Schaefer et al., 2018). The rs‐fMRI time‐series from 400 regions of interest (ROIs) were extracted. We constructed a 400 × 400 functional connectivity matrix based on Pearson correlations coefficients for each participant, performed *Z*‐transformation, and regressed covariates of age and sex.

Based on the Schaefer400 templates, the structural features of the cortical surface area were extracted from the T1w data, and the Kullback–Leibler (KL) divergence was calculated to construct structural connections. The KL divergence was calculated using the cortical surface area as follows: (1) The cortical surface area values of all the vertices corresponding to brain regions *i* and *k* of participant *j* were extracted. (2) The probability densities *f*_1_ and *f*_2_ of the surface area values of all vertices in the *i* and *k* brain regions of participant *j* were calculated, respectively (Equation (1)). (3) The divergence value of *f*_1_ was calculated with respect to *f*_2_ (Equation (3)) and *f*_2_ with respect to *f*_1_ (Equation (2)). To make the matrix symmetric, we constructed the symmetric KL divergence (Equations (3) and (4)). Similar calculations were performed for other participants and other brain regions. The KL divergence represents the morphological differences among brain regions: a smaller cortical morphology difference between two brain regions is represented by a smaller KL divergence. The KL divergence matrix was calculated as a structural connectome.

(1) $$f_{1}=\int f_{1}\left(x\right)dx\;f_{2}=\int f_{2}\left(x\right)dx$$

(2) $$\begin{array}{cc} \mathrm{KL}\left(f_{1}\|f_{2}\right)=\sum f_{1}\cdot \log\frac{f_{1}}{f_{2}} & \mathrm{KL}\left(f_{2}\|f_{1}\right)=\sum f_{2}\cdot \log\frac{f_{2}}{f_{1}} \end{array}$$

(3) $$\mathrm{KL}=\frac{\mathrm{KL}\left(f_{1}\|f_{2}\right)+\mathrm{KL}\left(f_{1}\|f_{2}\right)}{2}$$

(4) $$\mathrm{KL}\_\mathrm{adj}\left(i,k\right)=\mathrm{KL}\_\mathrm{adj}\left(k,i\right)=\mathrm{KL}$$

### 2.4 Gradient algorithm

**FIGURE 2:**
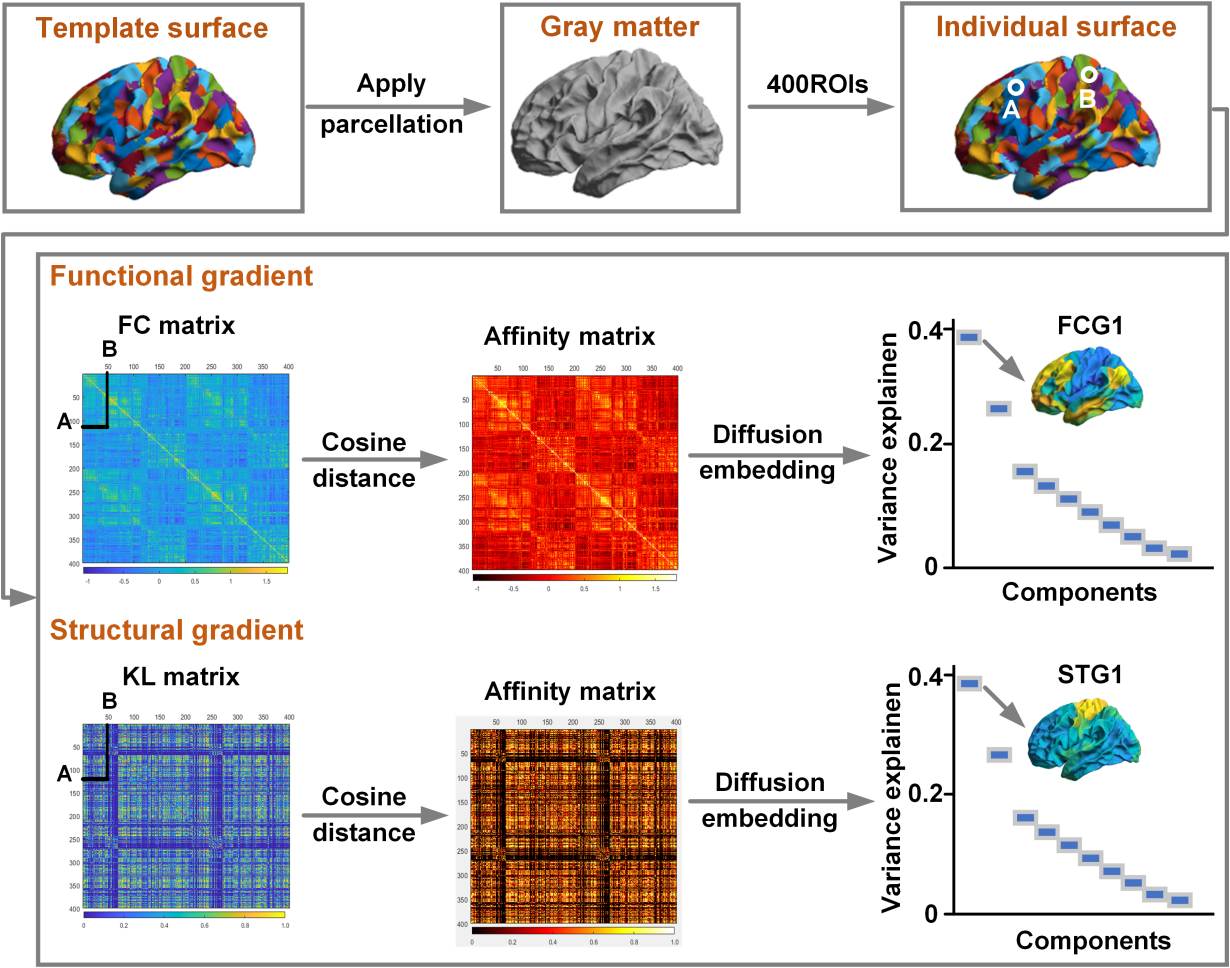
Pipeline for functional and structural gradient processing. The Schaefer400 template was mapped onto each individual surface to extract the functional and structural features in each parcellation. The KL and FC matrices were estimated using Pearson correlation coefficients. We transformed the connectivity matrix into an affinity matrix and obtained the components of the gradient by diffusion embedding. FC matrix, functional connectivity matrix; FCG1, principal functional gradient; KL matrix, Kullback–Leibler divergence matrix; STG1, principal structural gradient.

The processing pipeline of the gradient algorithm was based on the BrainSpace toolbox (de Wael et al., 2020). Based on the functional and structural matrix, we calculated a cosine similarity matrix that captures the similarity in connectivity between ROIs. Then, diffusion embedding (Margulies et al., 2016), a nonlinear manifold learning approach, was applied to capture the principal gradient component explaining the variance in the connectivity pattern. Compared to other nonlinear manifold learning techniques, the diffusion embedding algorithm is relatively robust to noise and computationally inexpensive. In brief, the algorithm estimates low‐dimensional embedding from a high‐dimensional connectome matrix. Notably, the algorithm is controlled by a single parameter α, which controls the influence of the density of sampling points on the manifold (*α* = 0, maximal influence; *α* = 1, no influence). As in a previous study, we set *α* = 0.5, a choice that retains the global relations between data points in the embedded space. The resulting gradient maps were further aligned across individuals using iterative Procrustes rotation (Hong et al., 2019). Our study focused on alterations in the principal gradient, which is closely associated with the neuronal microstructure and cognitive functions (Huntenburg et al., 2018; Margulies et al., 2016) (Figure 2).

Notably, the algorithm is controlled by a single parameter *α*, which controls the influence of the density of sampling points on the manifold (*α* = 0, maximal influence; *α* = 1, no influence). As in a previous study, we set *α* = 0.5, a choice that retains the global relations between data points in the embedded space. The resultant gradient maps were further aligned across individuals using iterative Procrustes rotation.

### 2.5 Support vector machine analysis

Using gradient features, a support vector machine (SVM) was constructed to discriminate patients from control participants. Network‐based gradient change analysis (Figure 4) revealed networks with differences between Pre_PD (before MIRT) and Post_PD (after MIRT) were obtained. The functional gradient (FCG) and structural gradient (STG) scores of healthy control participants and the Pre_PD group in these networks were extracted. Then, we used these FCG and STG scores as classification criteria. For comparison purposes, we estimated the classification ability of the SVM separately using the combined (FCG and STG) and individual (FCG or STG) features. Receiver operating characteristic (ROC) curve analysis was used to evaluate the classification models constructed from different sets of features. The evaluation index was the area under the curve (AUC) value.

### 2.6 Statistical analysis

Statistical analyses were performed using SPSS Statistics 26 at a threshold for statistical significance of *p* < .05, corrected with the false discovery rate (FDR). Descriptive statistics for continuous variables are presented as the means with standard deviations. Categorical variables are presented as counts and percentages. Univariate analyses of between‐group differences were performed using the independent‐samples *t* tests, paired‐samples *t* tests, or chi‐square tests, as appropriate. To verify that the network of association areas is related to movement, the Pearson correlation coefficient was used to assess the correlation between gradients and UPDRS‐III scores. Covariates, such as sex and age, were removed.

## 3 RESULTS

### 3.1 Demographic characteristics and clinical outcome

Seventy‐six patients and 34 healthy control participants were evaluated (Figure 1). The demographic characteristics and clinical outcomes of all participants are listed in Table 1. No significant differences were found in age, sex, or education between patients and control participants (independent‐samples *t* test and chi‐squared test, *p* > .05). According to the paired‐samples *t* test, patients' Pre_PD and Post_PD UPDRS‐III scores differed significantly (*p* < .05).

### 3.2 Functional and structural gradient patterns in PD

**FIGURE 3:**
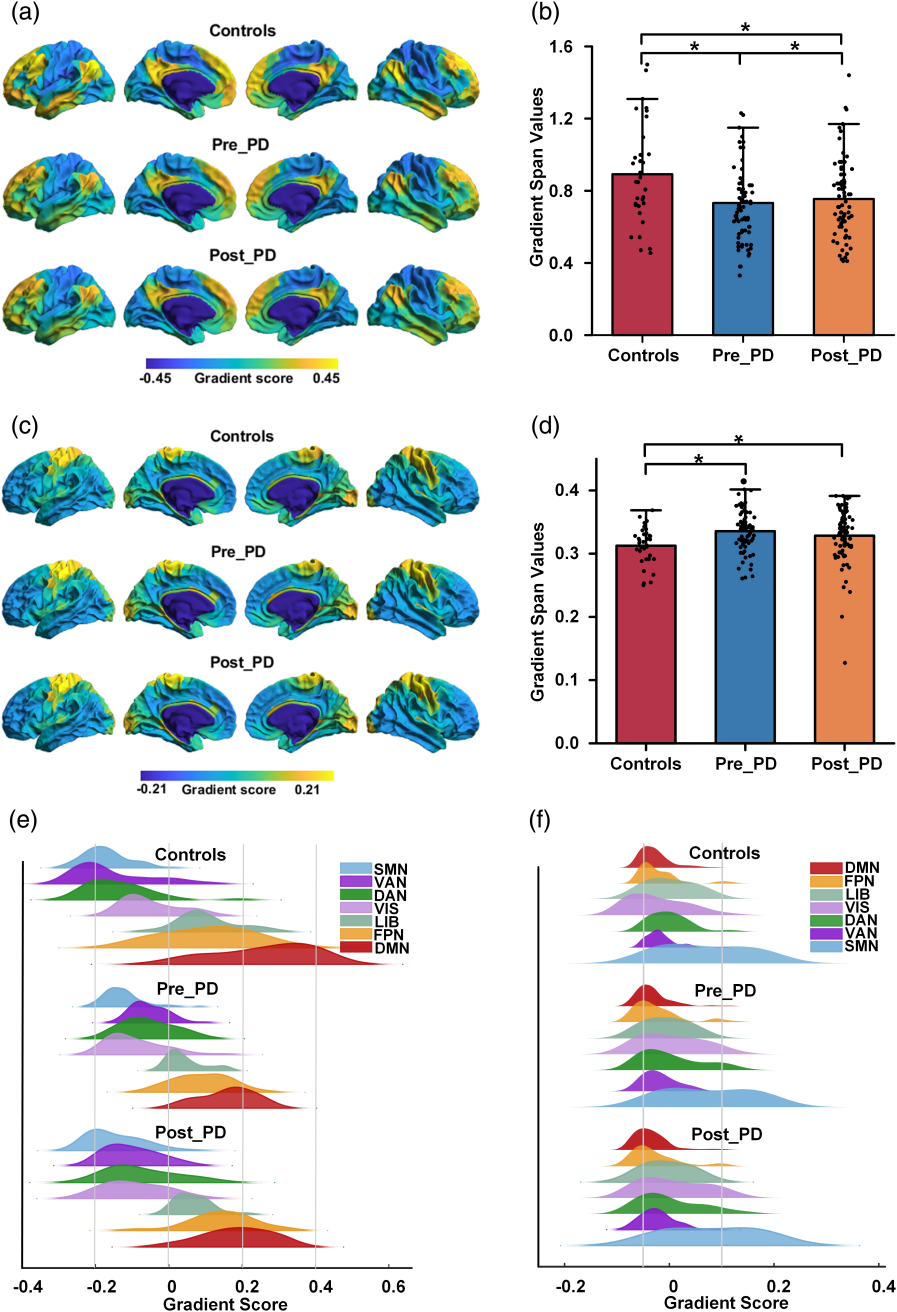
Functional and structural gradient mapping in patients with PD and control participants. (a) and (c) The principal functional and structural gradients of the controls and PD participants. Regions with similar connectivity patterns show similar colors. (b) and (d) Box scatter plots of the functional and structural gradient spans comparing the gradient scores of the group‐averaged maps of the PD patients and control participants. *, FDR‐corrected, *p* < .05. (e) and (f) Network‐based regional case–control difference analysis of the functional and structural gradient scores. DAN, dorsal attention network; DMN, default mode network; FPN, frontal parietal network; LIB, limbic network; Post_PD, PD patients after treatment; Pre_PD, PD patients before treatment; SMN, somatomotor network; VAN, ventral attention network; VIS, visual network.

The first gradient component (FCG, explained ratio > 30%, STG, explained ratio > 28%) was chosen as the principal gradient based on a scree plot (Figure S1). The principal FCG (FCG1) increased organizationally along a gradual axis from the visual network (VIS) or somatomotor network (SMN) to the association network (such as DMN, Figure 3a), while the principal STG (STG1) decreased along the somatomotor‐association axis (Figure 3c). The spatial patterns of the group‐averaged principal gradient maps were similar between patients and control participants. However, compared to those of the control participants, the FCG1 values were lower in the DMN, and the STG1 values were greater in the SMN in the PD patients. The Post_PD gradient differences were less than the Pre_PD differences.

We calculated the gradient span using the range of gradients across participants. The between‐group statistical comparisons showed that the FCG1 span in the PD group was significantly shorter than that in the control group (*, *p* < .05, Figure 3b). However, the Pre_PD and Post_PD STG1 span was greater than that in the control group, and the Post_PD span was shorter than the Pre_PD span (Figure 3d). The differences between the control group and the Pre_PD and Post_PD patient values were significant (*, *p* < .05), but that between the Pre_PD and Post_PD groups was not significant (*p* > .05).

A visual inspection of the network‐based gradient analysis results revealed that compared with control participants, the PD group had higher FCG1 scores in the SMN, DAN, and ventral attention network (VAN) and higher STG1 scores in the VIS and SMN but lower scores in the other networks (Figure 3e,f).

### 3.3 Altered functional and structural gradients in PD

**FIGURE 4:**
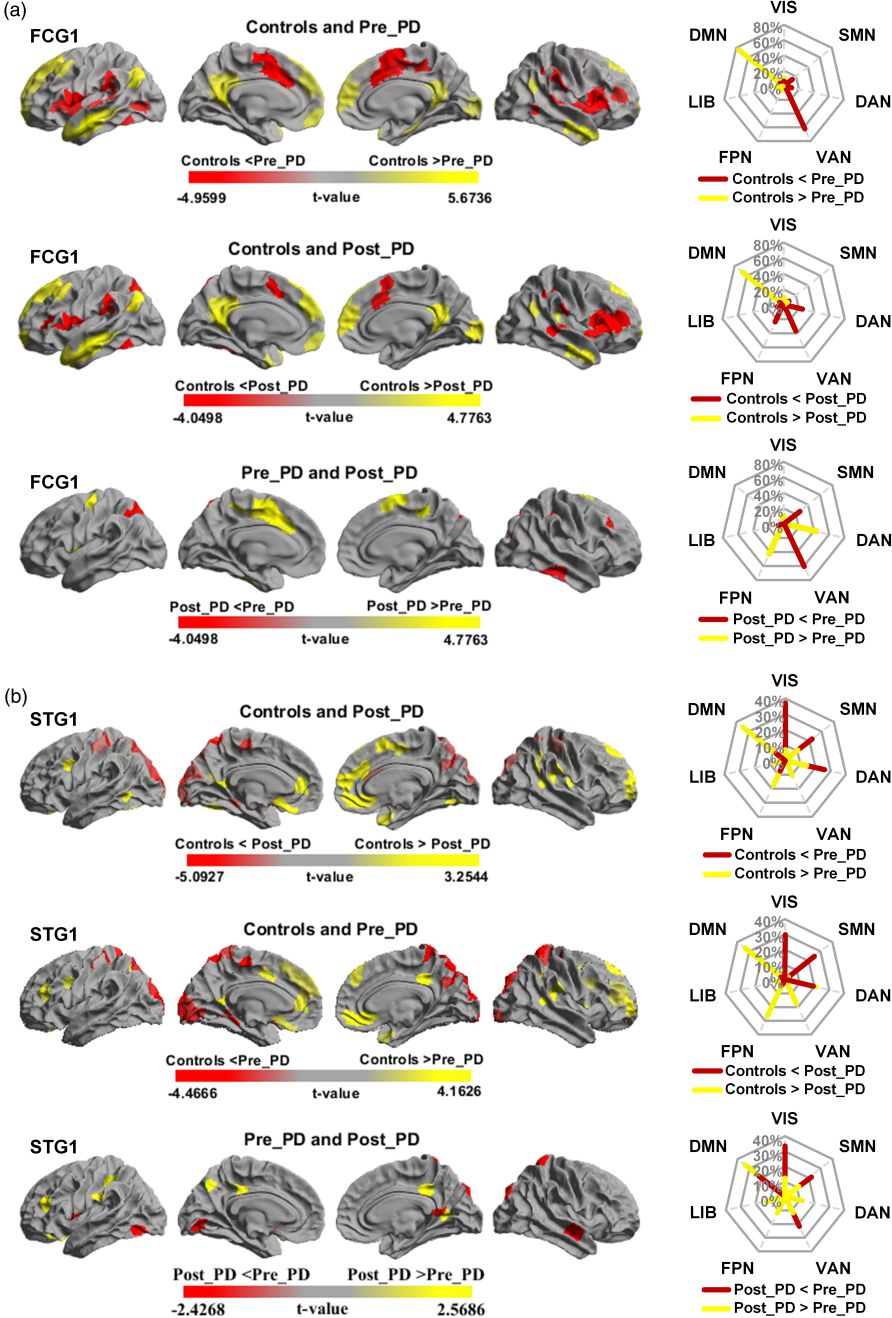
Statistical comparison of the gradient metrics. (a) and (b) Results of the statistical analysis of functional and structural gradients, respectively. The radar chart shows the proportions of different networks. Brain region statistical comparisons were made among the control participants, Pre_PD patients and Post_PD patients, with higher/lower values in PD patients presented as red/yellow colors. ROI‐level, FDR‐corrected, *p* < .05.

We conducted network‐based the between‐group and prepost statistical comparisons based on the network by using independent samples *t* tests (patients and control participants) and paired‐samples *t* tests (Pre_PD and Post_PD), respectively, across ROIs (*p* < .05). For FCG1, the PD group showed lower gradient scores mostly in the DMN and higher scores mainly in the VAN than did the control group (Figure 4a). Compared to the Post_PD group, the Pre_PD FCG1 scores were lower in the frontal parietal network (FPN) and DAN but higher in the SMN and VAN (Figure 4a). For STG1, the PD group showed lower gradient scores mostly in the DMN and higher scores distributed in the VIS, DAN, and SMN than did the control participants. In addition, the Post_PD group gradient scores in the FPN and DAN were lower (Figure 4b) than the Pre_PD group (Figure 4b). Compared to the Post_PD group, the Pre_PD group had lower gradient scores in the DMN and higher scores in the VIS and VAN (Tables S1 and S2).

### 3.4 Treatment modulates the gradient of networks

**FIGURE 5:**
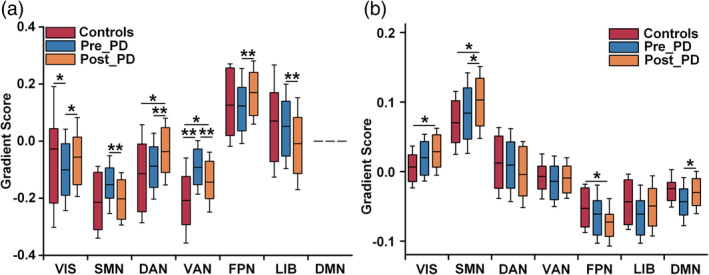
Group analysis of the gradients in different networks. (a) and (b) Functional and structural gradient‐score analysis of the different networks among the control participants, Pre_PD patients and Post_PD patients. Further statistical analysis revealed the networks that showed statistically significant improvement in PD patients following treatment. *, FDR‐corrected, *p* < .05; **, FDR‐corrected, *p* < .01.

The above *t* tests revealed those networks with differences between Pre_PD and Post_PD were obtained. For each network, we further compared the FCG1 and STG1 scores between patients (Pre_PD and Post_PD) and control participants by using the *t* tests (Figure 5). Next, the FCG1 and STG1 scores for these networks in the three groups were extracted to verify which functional and structural gradients were modulated by treatment. The comparison results showed that the networks with differences for FCG1 were the VIS, SMN, DAN, VAN, FPN, and limbic network (LIB), while those for STG1 were the VIS, SMN, and DMN (Figure 5, *, *p* < .05; **, *p* < .01, Table S3).

### 3.5 Classification analysis between PD patients and control participants

**FIGURE 6:**
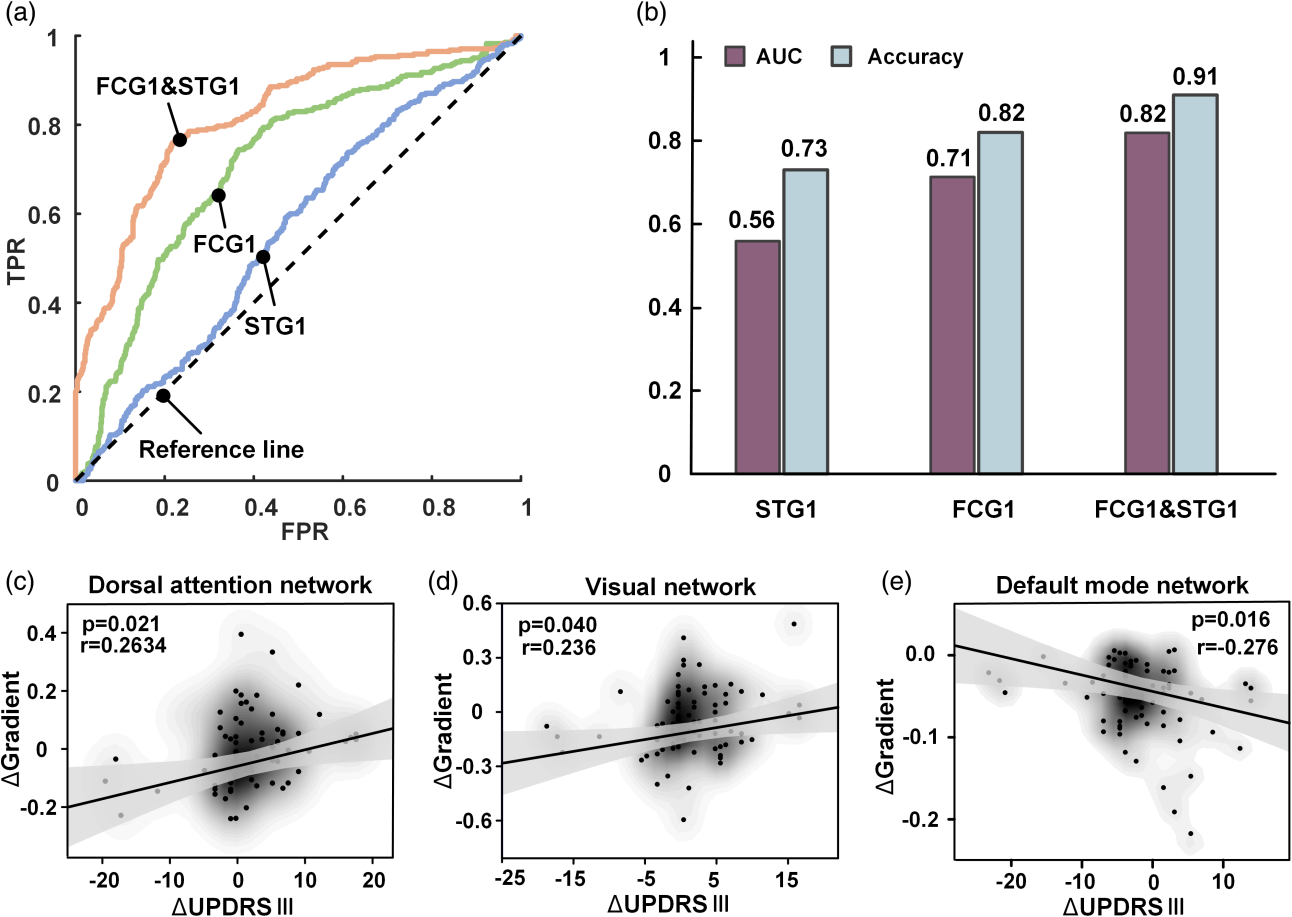
ROC curves and correlations between UPDRS‐III scores and gradient alterations. (a) ROC curves of different classification models. The different colors indicate the prediction curves of the model according to the combination of features of the principal functional and structural gradient (FCG1 and STG1, orange), in the features of the principal functional gradient (FCG1, green) and in the features of the principal structural gradient (STG1, blue). FPR, false positive rate; TPR, true positive rate. (b) The histogram shows the areas (purple) under the ROC curve (AUCs) and the classification accuracies (light blue) of the SVM. (c) and (d) Correlations between altered principal functional gradients and UPDRS‐III score changes from Pre_PD to Post_PD. (e) Correlations between altered principal structural gradients and UPDRS‐III score changes in Pre_PD and Post_PD. The translucent bands around the regression line represent the 95% confidence intervals for the regression estimates. The contour lines show the kernel density estimations between brain gradient values (FCG1/STG1 values) and clinical variables (UPDRS‐III). ΔGradient, |Gradient_Pre_PD_| − |Gradient_Post_PD_|; ΔUPDRS‐III, UPDRS‐III_Pre_PD_ − UPDRS‐III_Post_PD_.

The above network‐based gradient‐score analysis showed that the networks with differences in FCG1 included the VIS, SMN, DAN, VAN, FPN, and LIB networks, while the VIS, SMN, and DMN had differences in the STG1. We extracted the FCG1 and STG1 values of these networks as the feature vectors for SVM classification. The SVM classification models obtained through different feature values were evaluated by ROC curve analysis (Figure 6a). The results show that the combined feature of the FCG1 and STG1 signatures (accuracy = 0.91, AUC = 0.82) yielded better diagnostic performance than did the individual features (Figure 6b). Furthermore, the diagnostic effect for FCG1 (accuracy = 0.82, AUC = 0.71) was superior to that of STG1 (accuracy = 0.73, AUC = 0.56).

### 3.6 Correlations between treatment efficacy and clinical assessments

We obtained FCG1 and STG1 values from the networks with differences between Pre_PD and Post_PD and performed Pearson correlation analyses between altered gradients (|Gradient_Pre_PD_| − |Gradient_Post_PD_|) and clinical UPDRS‐III score changes (UPDRS‐III_Pre_PD_ − UPDRS‐III_Post_PD_), controlling for age and sex. The results showed that the UPDRS‐III score was correlated with alterations in the FCG (negative FCG1 values in DAN and VIS, |Gradient_Pre_PD_| > |Gradient_Post_PD_|, see Figure 5a) primarily in the DAN (*r* = 0.263, *p* = .022) and VIS (*r* = 0.236, *p* = .040), and with alterations in the STG1 (negative STG values in the DMN, |Gradient_Pre_PD_| < |Gradient_Post_PD_|, Figure 5b) primarily in the DMN (*r* = −0.276, *p* = .016) (Figure 6c–e). No correlations were detected for the other networks, such as FPN (*r* = 0.096, *p* = .409), SMN (*r* = 0.014, *p* = .902), or VAN (*r* = −0.160, *p* = .166).

## 4 DISCUSSION

This is the first study to investigate somatomotor‐association impairment and MIRT‐induced brain functional and structural alterations in PD patients by assessing functional and structural gradients. Abnormal functional and structural gradients were distributed across networks, including the primary sensorimotor network and the higher association network related to motor control and executive function. The combination of FCG1 and STG1 discriminated controls from patients well (accuracy >90%). Patients exhibited reduced gradient differences after treatment, which showed that networks with abnormal gradients can be modulated by treatment. We further found that FCG1 of the VIS and DAN and STG1 of the DMN were correlated with motor improvement (UPDRS‐III scores). These network changes included partial restoration of executive control and compensatory effects on attention information integration after treatment.

### 4.1 Altered functional and structural gradients in PD

The functional and structural gradient axes from sensorimotor to association regions represent the hierarchical organization of brain function and structure and can be used to explore sensorimotor disorders in PD patients. In PD patients, we found a narrower FCG1 span but a wider STG1 span, which may indicate a less differentiated functional connectivity pattern and greater similarity of cortical morphology between primary and association areas. The dissociation between the FCG1 and STG1 confirmed that cortical structure and function may not be organized in the same way across the whole brain (Vazquez‐Rodriguez et al., 2019). Treatment reduced the differences in the gradients of the brain networks. Notably, these networks are involved in multidomain functions, including primary processes (VIS, SMN) and higher‐order functions (DAN, VAN, and DMN). Among them, FCG1 of the VIS and DAN and STG1 of the DMN were correlated with motor improvement, and these networks are all related to motor control and coordination (Chen, Bedard, et al., 2021). Thus, there may be a potential link between gradient disruption and the concurrence of sensorimotor impairments in PD patients.

### 4.2 Compensatory and partial restoration mechanisms of rehabilitation treatment

The plasticity theory (Cauraugh & Summers, 2005) and neuropsychological functional connectivity models (Plaut, 1996) hypothesize that compensation and partial restitution are mechanisms of recovery. We demonstrated that the DAN exhibited a compensatory effect after treatment, which was reflected in higher FCG1 scores and was associated with motor improvement. The function of the DAN is to provide top‐down attentional orientation (Chen, Bedard, et al., 2021), which is closely related to spatial attention to facilitate attention guidance and motor planning of movement, and participates in the latter phase of motor learning (Eryurek et al., 2022). Compared to healthy control participants, several studies have shown that increased activation of DAN is associated with compensatory mechanisms for impaired motor function (Harrington et al., 2018). On the one hand, there is increased control of attention to compensate for affected movements (Maidan et al., 2019), such as improving gait and balance (Tard et al., 2015). On the other hand, enhanced motion perception compensates for impaired motor control (Halperin et al., 2021); for example, increasing position perception can improve motion control (Poudel et al., 2014). The compensation of the DAN in PD patients occurs within the network (Hartwigsen, 2018), which may include a stronger contribution of the remaining regions, a shift towards other regions in the network or recruitment of homologous regions in the other hemisphere (Hartwigsen, 2018).

In our study, the VIS exhibited partial restoration after treatment and was much more similar to that of the control participants than was the VIS at baseline. Visual impairment does occur in PD (Armstrong & Okun, 2020; Arrigo et al., 2017), and the visual cortex has been shown to be inhibited in patients with a motor subtype of PD, suggesting that visual information may affect motor symptoms (Hu et al., 2019; Suo et al., 2021). PD patients with visual impairment are unable to use sensory information accurately to plan and execute complex or new movements (Cucca et al., 2020). The functional restoration of the VIS in PD patients can improve the perception and processing of external motor information, thereby improving motor control (Shine et al., 2011). Moreover, visual‐motor feedback can help PD patients improve gait stability and walking speed (Gutierrez‐Herrera et al., 2017). Therefore, the functional restoration of the VIS has a positive effect on PD motor recovery. In addition, the DMN was partially restored in PD patients. The DMN is associated with motor planning and executive control (Smallwood et al., 2021), and is involved in top‐down control and regulation of information processing throughout the brain (Raichle, 2015). Studies have shown that decreased DMN activity correlates with the severity of PD dyskinesia. Dysfunction of the DMN in PD has been reported previously (Rektorova et al., 2012; Schindlbeck et al., 2021; van Eimeren et al., 2008). Importantly, we analysed the DMN from a structural perspective by using the STG, which provides a new perspective for understanding the role of the DMN in PD. Disruption of the DMN is a characteristic of PD, not only in PD patients with cognitive deficits (Pereira et al., 2015), but also in PD patients with unimpaired cognition (Sandrone & Catani, 2013). This finding suggested that the DMN may be altered in PD patients as a part of the pathological changes (Suo et al., 2021), consistent with the results that we obtained regarding structural gradient changes in the DMN.

Several issues need to be further addressed. First, PD patients were treated for only 2 weeks, and regular follow‐up investigations, which can be used to observe the changes in functional and structural gradients as disease progresses, were not conducted. Second, to further study the pathogenesis and rehabilitation mechanisms of PD, gene expression and transmitter receptor analyses are necessary to further identify and verify the relationships between the gradients and the transcriptome.

## 5 CONCLUSIONS

Our study revealed that FCG1 and STG1 are disrupted in PD patients, and an index combining FCG1 and STG1 could be used as a new diagnostic index for differentiating healthy controls and patients. In addition, we showed that the DAN, VIS, and DMN in PD patients undergo symptomatic improvement following treatment and explored the brain mechanisms by which these networks are associated with improved movement. Together, the results of this study provide insight into the coordination of structure and function in the hierarchical organization in PD patients.

## FUNDING INFORMATION

This work was supported by STI 2030‐Major Projects (grant number 2022ZD0208500); the National Natural Science Foundation of China (grant numbers U20A20191, 61727807, 82071912, 12104049, 82202291, 62373056); the Fundamental Research Funds for the Central Universities (grant number 2021CX11011); the Beijing Natural Science Foundation (23ISG023); the China Postdoctoral Science Foundation (grant numbers 2020TQ0040, 2022M710388).

## CONFLICT OF INTEREST STATEMENT

There are no conflicts of interest associated with this study.

## Supporting information


**Appendix S1:** Supporting information.

## Data Availability

The sensitive nature of patients' data do not permit open data sharing. The clinical and neuroimaging data used in the current paper are available from the corresponding author (yantianyi@bit.edu.cn, fangboyanv@ccmu.edu.cn) on request.
