# Supplementary material for: Functional and structural gradients reveal atypical hierarchical organization of Parkinson's disease
Source: Hum Brain Mapp. 2024 Mar 15;45(4):e26647. doi: 10.1002/hbm.26647 (PMC10941507; doi:10.1002/hbm.26647)
Supplement: Supplementary file 1 — Appendix S1: Supporting information. [file HBM-45-e26647-s001.docx]

# Supplementary Material

**Appendix 1:** Multidisciplinary Intensive Rehabilitation therapy (MIRT)

**Appendix 2:** Data Processing

**Supplementary Table 1.** Networks significant between-group differences in principal functional gradient (FCG1)

**Supplementary Table 2.** Networks significant between-group differences in principal structural gradient (STG1)

**Supplementary Table 3.** Networks with significant between-group differences in principal functional and structural gradient

**Supplementary Figure 1.** Scree plot of the first 20 components

**Appendix 1: Multidisciplinary Intensive Rehabilitation therapy (MIRT)**

The first part was one-on-one physical therapy that included cardiovascular warm-up exercises, active-passive exercises aimed at improving joint range of motion, abdominal stretched, strengthened the paravertebral muscles, posture adjusted and controlled of movement. The second part training was performed on the C-Mill treadmill (a treadmill with augmented reality technology, visual cues, auditory cues and feedback) using various instruments to improve gait and balance. Once in the morning and afternoon every day, each training 30 minutes. The third part is aerobic exercise. Aerobic training was performed on a power bike with feedback for 30 minutes. The fourth part is speech therapy. Including three possible interventions: (1) Counseling patients and caregivers on how to properly manage speech and swallowing problems; (2) Swallowing training, including dietary monitoring and learning proper ways to take in food and fluids; (3) Speech therapy for motor dysarthria (Breathing training to relax and relieve speech pressure; Facial movement to improve the range of facial expression and mouth movement; Exercises to improve vocal pronunciation and speech rhythm). During all activities, exercise intensity was maintained at 70 to 80 percent of maximum heart rate. When patients left the hospital, Specialized rehabilitation therapists introduced the Parkinson's chronic disease management platform to patients, and explain the operating methods to the patient, to ensure that patients can independently use the platform for home rehabilitation training after discharge. Patients were required to clock, in after completing daily video course training, to motivate and supervise patients to carry out home rehabilitation training. Through explicit and implicit learning strategies, MIRT facilitates gait correction, improves balance, increases one's ability to perform aerobic exercise, and entails cognitive motor therapy through which patients relearn motor dysfunction that has occurred due to disease.

**Appendix 2: Data Processing**

Structural MRI data processing was based on FreeSurfer version 6.0.1 software ([https: //surfer.nmr.mgh.harvard.edu/](https://surfer.nmr.mgh.harvard.edu/)) and ANTs (v5.0.9, <https://www.nitrc.org>/projects/ants). Briefly, processing of the T1w image included intensity nonuniformity correction, skull stripping, cortical extraction and segmentation of cortical white and grey matter. Brain surfaces were reconstructed using recon-all (FreeSurfer). Volume-based spatial normalization to one standard space (MNI152 NLin2009cAsym) was performed through nonlinear registration with ANTs registration, using brain-extracted versions of both the T1w reference and the T1w template.

The rs-fMRI data processing pipeline was built using DPABISurf (a surface-based resting-state fMRI data analysis toolbox, <http://www.rfmri.org/dpabi>). The DPABISurf pipeline first converts the fMRI data into BIDS format, and then calls fMRIPprep docker to preprocess the functional MRI data, which integrates FreeSurfer, ANTs, FSL and AFNI. With fMRIPprep, the data is processed into FreeSurfer fsaverage5 surface space. DPABISurf can be used to further performs nuisance covariate regression (including independent component analysis-automatic removal of motion artifacts (ICA-AROMA)) on the surface-based data and then calculate the functional connectivity. Image processing included removing the first 10 frames, slice-timing correction, and motion correction using rigid body translation and rotation. The functional image was then coregistered to the T1w reference using bbregister (FreeSurfer) which implements boundary-based registration. Coregistration was configured with six degrees of freedom. The rs-fMRI surface data were resampled onto the fsaverage5, and applied surface-based smoothing (5-mm full width at half maximum) was applied. MRI quality control was complemented by assessment of the signal-to-noise ratio and visual scoring of surface extractions for structural MRI.

**Supplementary Table 1. Networks significant between-group differences in principal** **functional gradient (FCG1)**

| **Group** | **Label** | **Brain region** | **Network name** |
| --- | --- | --- | --- |
| Controls < Pre_PD | 17 | 7Networks_LH_Vis_17 | Visual network |
|  | 33  37  40  56  237  238  254 | 7Networks_LH_SomMot_2  7Networks_LH_SomMot_6  7Networks_LH_SomMot_9  7Networks_LH_SomMot_25  7Networks_RH_SomMot_7  7Networks_RH_SomMot_8  7Networks_RH_SomMot_24 | Somatomotor  network |
|  | 70  77  272  281  290 | 7Networks_LH_DorsAttn_Post_2  7Networks_LH_DorsAttn_Post_9  7Networks_RH_DorsAttn_Post_2  7Networks_RH_DorsAttn_Post_11  7Networks_RH_DorsAttn_FEF_1 | Dorsal attention network |
|  | 92  95  98  99  101  102  103  104  105  107  108  110  298  299  303  305  306  307  308  309  311  312  313  314  316  317 | 7Networks_LH_SalVentAttn_ParOper_1  7Networks_LH_SalVentAttn_ParOper_4  7Networks_LH_SalVentAttn_FrOperIns_2  7Networks_LH_SalVentAttn_FrOperIns_3  7Networks_LH_SalVentAttn_FrOperIns_5  7Networks_LH_SalVentAttn_FrOperIns_6  7Networks_LH_SalVentAttn_FrOperIns_7  7Networks_LH_SalVentAttn_FrOperIns_8  7Networks_LH_SalVentAttn_FrOperIns_9  7Networks_LH_SalVentAttn_Med_1  7Networks_LH_SalVentAttn_Med_2  7Networks_LH_SalVentAttn_Med_4  7Networks_RH_SalVentAttn_TempOccPar_5  7Networks_RH_SalVentAttn_TempOccPar_6  7Networks_RH_SalVentAttn_FrOperIns_2  7Networks_RH_SalVentAttn_FrOperIns_4  7Networks_RH_SalVentAttn_FrOperIns_5  7Networks_RH_SalVentAttn_FrOperIns_6  7Networks_RH_SalVentAttn_FrOperIns_7  7Networks_RH_SalVentAttn_FrOperIns_8  7Networks_RH_SalVentAttn_Med_1  7Networks_RH_SalVentAttn_Med_2  7Networks_RH_SalVentAttn_Med_3  7Networks_RH_SalVentAttn_Med_4  7Networks_RH_SalVentAttn_Med_6  7Networks_RH_SalVentAttn_Med_7 | Ventral attention network |
|  | 378 | 7Networks_RH_Default_PFCv_4 | Default mode network |
| Controls > Pre_PD | 20  215  217 | 7Networks_LH_Vis_20  7Networks_RH_Vis_15  7Networks_RH_Vis_17 | Visual network |
|  | 118  124  327  331 | 7Networks_LH_Limbic_OFC_5  7Networks_LH_Limbic_TempPole_6  7Networks_RH_Limbic_TempPole_3  7Networks_RH_Limbic_TempPole_7 | Limbic network |
|  | 345  347 | 7Networks_RH_Cont_PFCl_5  7Networks_RH_Cont_PFCl_7 | Frontal parietal control network |
|  | 149  150  152  154  155  162  164  165  168  176  178  179  180  182  183  184  187  188  190  191  192  193  195  196  364  367  368  380  382  385  389  392  393  395 | 7Networks_LH_Default_Temp_1  7Networks_LH_Default_Temp_2  7Networks_LH_Default_Temp_4  7Networks_LH_Default_Temp_6  7Networks_LH_Default_Temp_7  7Networks_LH_Default_Par_4  7Networks_LH_Default_Par_6  7Networks_LH_Default_Par_7  7Networks_LH_Default_PFC_3  7Networks_LH_Default_PFC_11  7Networks_LH_Default_PFC_13  7Networks_LH_Default_PFC_14  7Networks_LH_Default_PFC_15  7Networks_LH_Default_PFC_17  7Networks_LH_Default_PFC_18  7Networks_LH_Default_PFC_19  7Networks_LH_Default_PFC_22  7Networks_LH_Default_PFC_23  7Networks_LH_Default_pCunPCC_1  7Networks_LH_Default_pCunPCC_2  7Networks_LH_Default_pCunPCC_3  7Networks_LH_Default_pCunPCC_4  7Networks_LH_Default_pCunPCC_6  7Networks_LH_Default_pCunPCC_7  7Networks_RH_Default_Par_3  7Networks_RH_Default_Temp_1  7Networks_RH_Default_Temp_2  7Networks_RH_Default_PFCdPFCm_2  7Networks_RH_Default_PFCdPFCm_4  7Networks_RH_Default_PFCdPFCm_7  7Networks_RH_Default_PFCdPFCm_11  7Networks_RH_Default_pCunPCC_1  7Networks_RH_Default_pCunPCC_2  7Networks_RH_Default_pCunPCC_4 | Default mode network |
| Controls < Post_PD | 40  237 | 7Networks_LH_SomMot_9  7Networks_RH_SomMot_7 | Somatomotor  network |
|  | 69  70  74  75  77  81  278  281 | 7Networks_LH_DorsAttn_Post_1  7Networks_LH_DorsAttn_Post_2  7Networks_LH_DorsAttn_Post_6  7Networks_LH_DorsAttn_Post_7  7Networks_LH_DorsAttn_Post_9  7Networks_LH_DorsAttn_Post_13  7Networks_RH_DorsAttn_Post_8  7Networks_RH_DorsAttn_Post_11 | Dorsal attention network |
|  | 92  95  101  102  103  105  110  298  303  306  309  311  314 | 7Networks_LH_SalVentAttn_ParOper_1  7Networks_LH_SalVentAttn_ParOper_4  7Networks_LH_SalVentAttn_FrOperIns_5  7Networks_LH_SalVentAttn_FrOperIns_6  7Networks_LH_SalVentAttn_FrOperIns_7  7Networks_LH_SalVentAttn_FrOperIns_9  7Networks_LH_SalVentAttn_Med_4  7Networks_RH_SalVentAttn_TempOccPar_5  7Networks_RH_SalVentAttn_FrOperIns_2  7Networks_RH_SalVentAttn_FrOperIns_5  7Networks_RH_SalVentAttn_FrOperIns_8  7Networks_RH_SalVentAttn_Med_1  7Networks_RH_SalVentAttn_Med_4 | Ventral attention network |
|  | 132  136  335  336  344  345  346  347 | 7Networks_LH_Cont_Par_6  7Networks_LH_Cont_PFCl_2  7Networks_RH_Cont_Par_4  7Networks_RH_Cont_Par_5  7Networks_RH_Cont_PFCl_4  7Networks_RH_Cont_PFCl_5  7Networks_RH_Cont_PFCl_6  7Networks_RH_Cont_PFCl_7 | Frontal parietal control network |
|  | 374  378 | 7Networks_RH_Default_Temp_8  7Networks_RH_Default_PFCv_4 | Default mode network |
| Controls > Post_PD | 20  215  217 | 7Networks_LH_Vis_20  7Networks_RH_Vis_15  7Networks_RH_Vis_17 | Visual network |
|  | 240  255 | 7Networks_RH_SomMot_10  7Networks_RH_SomMot_25 | Somatomotor  network |
|  | 118  120  124 | 7Networks_LH_Limbic_OFC_5  7Networks_LH_Limbic_TempPole_2  7Networks_LH_Limbic_TempPole_6 | Limbic network |
|  | 149  150  152  154  155  162  164  168  178  179  182  183  184  187  188  190  191  192  193  195  196  364  367  368  380  382  385  389  392  393  395 | 7Networks_LH_Default_Temp_1  7Networks_LH_Default_Temp_2  7Networks_LH_Default_Temp_4  7Networks_LH_Default_Temp_6  7Networks_LH_Default_Temp_7  7Networks_LH_Default_Par_4  7Networks_LH_Default_Par_6  7Networks_LH_Default_PFC_3  7Networks_LH_Default_PFC_13  7Networks_LH_Default_PFC_14  7Networks_LH_Default_PFC_17  7Networks_LH_Default_PFC_18  7Networks_LH_Default_PFC_19  7Networks_LH_Default_PFC_22  7Networks_LH_Default_PFC_23  7Networks_LH_Default_pCunPCC_1  7Networks_LH_Default_pCunPCC_2  7Networks_LH_Default_pCunPCC_3  7Networks_LH_Default_pCunPCC_4  7Networks_LH_Default_pCunPCC_6  7Networks_LH_Default_pCunPCC_7  7Networks_RH_Default_Par_3  7Networks_RH_Default_Temp_1  7Networks_RH_Default_Temp_2  7Networks_RH_Default_PFCdPFCm_2  7Networks_RH_Default_PFCdPFCm_4  7Networks_RH_Default_PFCdPFCm_7  7Networks_RH_Default_PFCdPFCm_11  7Networks_RH_Default_pCunPCC_1  7Networks_RH_Default_pCunPCC_2  7Networks_RH_Default_pCunPCC_4 | Default mode network |
| Post_PD < Pre_PD | 49  54  56  57 | 7Networks_LH_SomMot_18  7Networks_LH_SomMot_23  7Networks_LH_SomMot_25  7Networks_LH_SomMot_26 | Somatomotor  network |
|  | 104  107  108  110  314  316  317  318 | 7Networks_LH_SalVentAttn_FrOperIns_8  7Networks_LH_SalVentAttn_Med_1  7Networks_LH_SalVentAttn_Med_2  7Networks_LH_SalVentAttn_Med_4  7Networks_RH_SalVentAttn_Med_4  7Networks_RH_SalVentAttn_Med_6  7Networks_RH_SalVentAttn_Med_7  7Networks_RH_SalVentAttn_Med_8 | Ventral attention network |
|  | 123 | 7Networks_LH_Limbic_TempPole_5 | Limbic network |
| Post_PD > Pre_PD | 227 | 7Networks_RH_Vis_27 | Visual network |
|  | 75  78  81  277 | 7Networks_LH_DorsAttn_Post_7  7Networks_LH_DorsAttn_Post_10  7Networks_LH_DorsAttn_Post_13  7Networks_RH_DorsAttn_Post_7 | Dorsal attention network |
|  | 335  337  338  351 | 7Networks_RH_Cont_Par_4  7Networks_RH_Cont_Par_6  7Networks_RH_Cont_Temp_1  7Networks_RH_Cont_PFCl_11 | Frontal parietal control network |

**Supplementary Table 2. Networks significant between-group differences in principal structural gradient (STG1)**

| **Group** | **Label** | **Brain region** | **Network name** |
| --- | --- | --- | --- |
| Controls < Pre_PD | 7  9  15  24  25  29  31  223  229  230 | 7Networks_LH_Vis_7  7Networks_LH_Vis_9  7Networks_LH_Vis_15  7Networks_LH_Vis_24  7Networks_LH_Vis_25  7Networks_LH_Vis_29  7Networks_LH_Vis_31  7Networks_RH_Vis_23  7Networks_RH_Vis_29  7Networks_RH_Vis_30 | Visual network |
|  | 51  52  55  60  253  263 | 7Networks_LH_SomMot_20  7Networks_LH_SomMot_21  7Networks_LH_SomMot_24  7Networks_LH_SomMot_29  7Networks_RH_SomMot_23  7Networks_RH_SomMot_33 | Somatomotor  network |
|  | 81  83  84  277  285  288  289 | 7Networks_LH_DorsAttn_Post_13  7Networks_LH_DorsAttn_Post_15  7Networks_LH_DorsAttn_Post_16  7Networks_RH_DorsAttn_Post_7  7Networks_RH_DorsAttn_Post_15  7Networks_RH_DorsAttn_Post_18  7Networks_RH_DorsAttn_Post_19 | Dorsal attention network |
|  | 144  356 | 7Networks_LH_Cont_pCun_1  7Networks_RH_Cont_pCun_1 | Frontal parietal control network |
|  | 384  396 | 7Networks_RH_Default_PFCdPFCm_6  7Networks_RH_Default_pCunPCC_5 | Default mode network |
| Controls > Pre_PD | 3  206 | 7Networks_LH_Vis_3  7Networks_RH_Vis_6 | Visual network |
|  | 235  240  254 | 7Networks_RH_SomMot_5  7Networks_RH_SomMot_10  7Networks_RH_SomMot_24 | Somatomotor  networ |
|  | 71  73  77  281 | 7Networks_LH_DorsAttn_Post_3  7Networks_LH_DorsAttn_Post_5  7Networks_LH_DorsAttn_Post_9  7Networks_RH_DorsAttn_Post_11 | Dorsal attention network |
|  | 295  298  314 | 7Networks_RH_SalVentAttn_TempOccPar_2  7Networks_RH_SalVentAttn_TempOccPar_5  7Networks_RH_SalVentAttn_Med_4 | Ventral attention network |
|  | 117  329 | 7Networks_LH_Limbic_OFC_4  7Networks_RH_Limbic_TempPole_5 | Limbic network |
|  | 130  141  337  343  360 | 7Networks_LH_Cont_Par_4  7Networks_LH_Cont_PFCl_7  7Networks_RH_Cont_Par_6  7Networks_RH_Cont_PFCl_3  7Networks_RH_Cont_PFCmp_1 | Frontal parietal control network |
|  | 167  174  190  191  365  376  379  382  383  389 | 7Networks_LH_Default_PFC_2  7Networks_LH_Default_PFC_9  7Networks_LH_Default_pCunPCC_1  7Networks_LH_Default_pCunPCC_2  7Networks_RH_Default_Par_4  7Networks_RH_Default_PFCv_2  7Networks_RH_Default_PFCdPFCm_1  7Networks_RH_Default_PFCdPFCm_4  7Networks_RH_Default_PFCdPFCm_5  7Networks_RH_Default_PFCdPFCm_11 | Default mode network |
| Controls < Post_PD | 7  9  11  13  15  18  24  29  213  223  229  230 | 7Networks_LH_Vis_7  7Networks_LH_Vis_9  7Networks_LH_Vis_11  Networks_LH_Vis_13  7Networks_LH_Vis_15  7Networks_LH_Vis_18  7Networks_LH_Vis_24  7Networks_LH_Vis_29  7Networks_RH_Vis_13  7Networks_RH_Vis_23  7Networks_RH_Vis_29  7Networks_RH_Vis_30 | Visual network |
|  | 51  55  60  63  258  262  263  264  265  267 | 7Networks_LH_SomMot_20  7Networks_LH_SomMot_24  7Networks_LH_SomMot_29  7Networks_LH_SomMot_32  7Networks_RH_SomMot_28  7Networks_RH_SomMot_32  7Networks_RH_SomMot_33  7Networks_RH_SomMot_34  7Networks_RH_SomMot_35  7Networks_RH_SomMot_37 | Somatomotor  network |
|  | 81  83  84  85  277  285  288  289 | 7Networks_LH_DorsAttn_Post_13  7Networks_LH_DorsAttn_Post_15  7Networks_LH_DorsAttn_Post_16  7Networks_LH_DorsAttn_Post_17  7Networks_RH_DorsAttn_Post_7  7Networks_RH_DorsAttn_Post_15  7Networks_RH_DorsAttn_Post_18  7Networks_RH_DorsAttn_Post_19 | Dorsal attention network |
|  | 356 | 7Networks_RH_Cont_pCun_1 | Frontal parietal control network |
|  | 396 | 7Networks_RH_Default_pCunPCC_5 | Default mode network |
| Controls > Post_PD | 240 | 7Networks_RH_SomMot_10 | Somatomotor  network |
|  | 73  75  77  275  278  281  291 | 7Networks_LH_DorsAttn_Post_5  7Networks_LH_DorsAttn_Post_7  7Networks_LH_DorsAttn_Post_9  7Networks_RH_DorsAttn_Post_5  7Networks_RH_DorsAttn_Post_8  7Networks_RH_DorsAttn_Post_11  7Networks_RH_DorsAttn_FEF_2 | Dorsal attention network |
|  | 106  108  295  298  308  310 | 7Networks_LH_SalVentAttn_PFCl_1  7Networks_LH_SalVentAttn_Med_2  7Networks_RH_SalVentAttn_TempOccPar_2  7Networks_RH_SalVentAttn_TempOccPar_5  7Networks_RH_SalVentAttn_FrOperIns_7  7Networks_RH_SalVentAttn_PFCl_1 | Ventral attention network |
|  | 117  329 | 7Networks_LH_Limbic_OFC_4  7Networks_RH_Limbic_TempPole_5 | Limbic network |
|  | 130  139  141  148  337  343  345  348  350 | 7Networks_LH_Cont_Par_4  7Networks_LH_Cont_PFCl_5  7Networks_LH_Cont_PFCl_7  7Networks_LH_Cont_PFCmp_1  7Networks_RH_Cont_Par_6  7Networks_RH_Cont_PFCl_3  7Networks_RH_Cont_PFCl_5  7Networks_RH_Cont_PFCl_8  7Networks_RH_Cont_PFCl_10 | Frontal parietal control network |
|  | 174  178  190  376  379  387  389  397 | 7Networks_LH_Default_PFC_9  7Networks_LH_Default_PFC_13  7Networks_LH_Default_pCunPCC_1  7Networks_RH_Default_PFCv_2  7Networks_RH_Default_PFCdPFCm_1  7Networks_RH_Default_PFCdPFCm_9  7Networks_RH_Default_PFCdPFCm_11  7Networks_RH_Default_pCunPCC_6 | Default mode network |
| Post_PD < Pre_PD | 8  11  229 | 7Networks_LH_Vis_8  7Networks_LH_Vis_11  7Networks_RH_Vis_29 | Visual network |
|  | 48  267 | 7Networks_LH_SomMot_17  7Networks_RH_SomMot_37 | Somatomotor  network |
|  | 102  104 | 7Networks_LH_SalVentAttn_FrOperIns_6  7Networks_LH_SalVentAttn_FrOperIns_8 | Ventral attention network |
|  | 370  392 | 7Networks_RH_Default_Temp_4  7Networks_RH_Default_pCunPCC_1 | Default mode network |
| Post_PD > Pre_PD | 220 | 7Networks_RH_Vis_20 | Visual network |
|  | 79 | 7Networks_LH_DorsAttn_Post_11 | Dorsal attention network |
|  | 94 | 7Networks_LH_SalVentAttn_ParOper_3 | Ventral attention network |
|  | 115 | 7Networks_LH_Limbic_OFC_2 | Limbic network |
|  | 139 | 7Networks_LH_Cont_PFCl_5 | Frontal parietal control network |
|  | 197  397  199 | 7Networks_LH_Default_pCunPCC_8  7Networks_RH_Default_pCunPCC_6  7Networks_LH_Default_pCunPCC_10 | Default mode network |

**Supplementary Table 3. Networks with significant between-group differences in principal functional and structural gradient**

|  | **Controls vs. Pre_PD**  ***p* -value** | | **Controls vs. Post_PD**  ***p* -value** | | **Pre_PD vs. Post_PD**  ***p* -value** | |
| --- | --- | --- | --- | --- | --- | --- |
| **Network** | **FCG1** | **STG1** | **FCG1** | **STG1** | **FCG1** | **STG1** |
| VIS | 0.137 | 0.332 | 0.566 | **0.010** | **0.038** | 0.067 |
| SMN | **0.021** | 0.470 | 0.573 | **0.014** | **0.003** | **0.026** |
| DAN | 0.517 | 0.895 | **0.033** | 0.178 | **0.005** | 0.067 |
| VAN | **0.000** | 0.470 | **0.033** | 0.711 | **0.003** | 0.226 |
| FPN | 0.915 | 0.470 | 0.124 | **0.014** | **0.006** | 0.067 |
| LIB | 0.696 | 0.470 | 0.056 | 0.574 | **0.006** | 0.067 |
| DMN | — | 0.895 | — | 0.479 | — | **0.042** |

**Supplementary Figure 1. Scree plot of the first 20 components. (A)** and **(B)** The averaged explained ratio of the first 20 diffusion embedding components of the functional and structural gradient in the controls and PD patients. The principal functional gradient explained 31.8% of the total connectivity variance (controls, 34.6%; Pre_PD, 30.6%; Post_PD, 30.2%). The principal structural gradient explained 27.7% of the total connectivity variance (controls, 27.2%; Pre_PD, 27.9%; Post_PD, 27.9%).

| **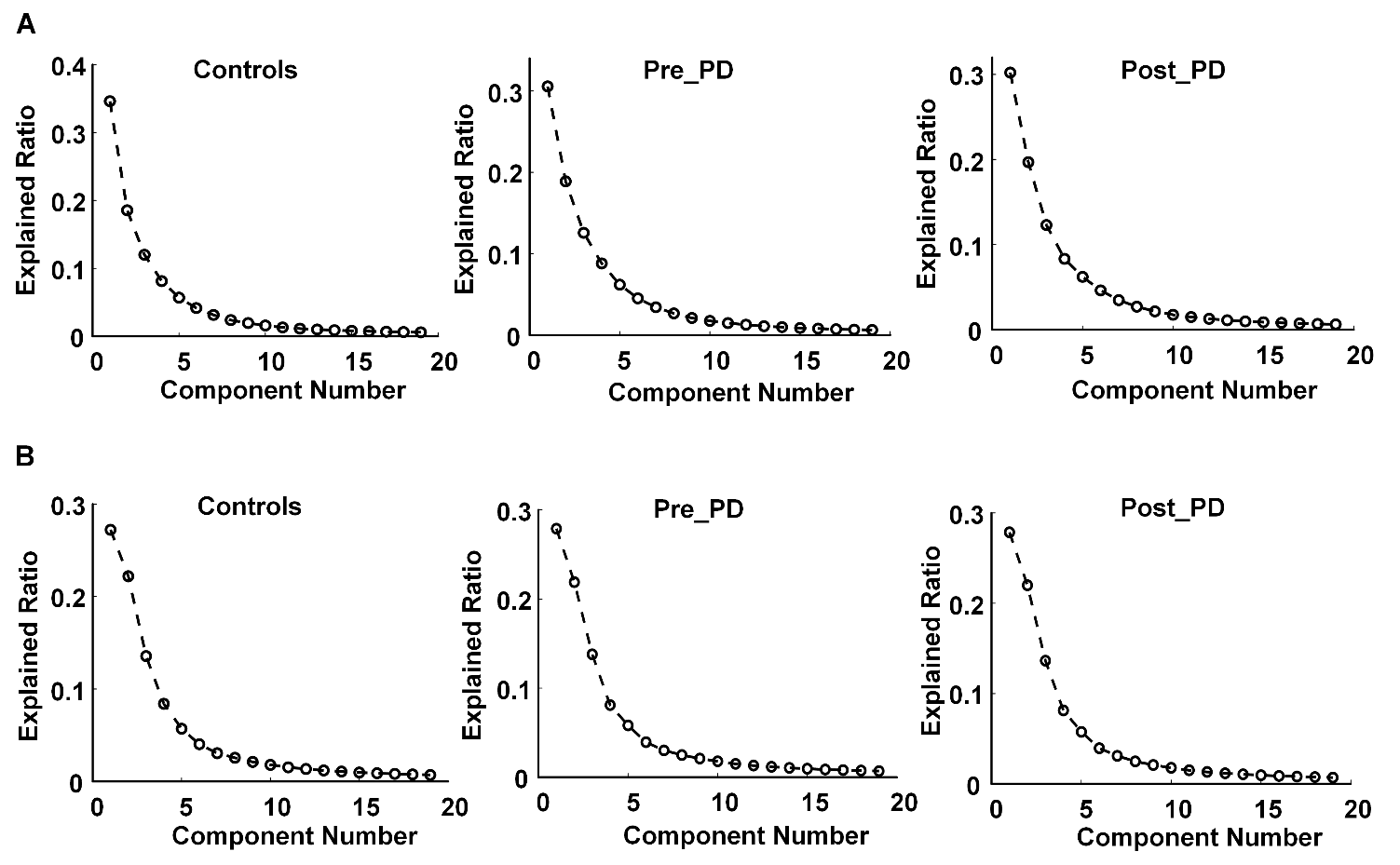** |
| --- |
